# No Sensory Compensation for Olfactory Memory: Differences between Blind and Sighted People

**DOI:** 10.3389/fpsyg.2017.02127

**Published:** 2017-12-08

**Authors:** Agnieszka Sorokowska, Maciej Karwowski

**Affiliations:** ^1^Smell and Taste Clinic, Department of Otorhinolaryngology, Technische Universität Dresden, Dresden, Germany; ^2^Institute of Psychology, University of Wrocław, Wrocław, Poland

**Keywords:** olfactory memory, olfaction, blindness, visual impairment, aging, sensory compensation

## Abstract

Blindness can be a driving force behind a variety of changes in sensory systems. When vision is missing, other modalities and higher cognitive functions can become hyper-developed through a mechanism called sensory compensation. Overall, previous studies suggest that olfactory memory in blind people can be better than that of the sighted individuals. Better performance of blind individuals in other-sensory modalities was hypothesized to be a result of, among others, intense perceptual training. At the same time, if the superiority of blind people in olfactory abilities indeed results from training, their scores should not decrease with age to such an extent as among the sighted people. Here, this hypothesis was tested in a large sample of 94 blind individuals. Olfactory memory was assessed using the Test for Olfactory Memory, comprising episodic odor recognition (discriminating previously presented odors from new odors) and two forms of semantic memory (cued and free identification of odors). Regarding episodic olfactory memory, we observed an age-related decline in correct hits in blind participants, but an age-related increase in false alarms in sighted participants. Further, age moderated the between-group differences for correct hits, but the direction of the observed effect was contrary to our expectations. The difference between blind and sighted individuals younger than 40 years old was non-significant, but older sighted individuals outperformed their blind counterparts. In conclusion, we found no positive effect of visual impairment on olfactory memory. We suggest that daily perceptual training is not enough to increase olfactory memory function in blind people.

## Introduction

Blindness can be a driving force behind a variety of changes in sensory systems. When vision is missing, other modalities and higher cognitive functions can become hyper-developed (Pascual-Leone et al., 2005) through a mechanism called sensory compensation (Kupers and Ptito, 2014). Better performance of blind individuals in other-sensory modalities was hypothesized to be a result of either intense perceptual training (Gagnon et al., 2015), reorganization of various brain areas (e.g., the occipital cortex, Leclerc et al., 2000), or a combination of both of these mechanisms (Röder and Rösler, 2003).

Among many possible types of compensation, increased memory ability of blind people has been the focus of several studies. Visual impairment has been found to be related to better general memory (Amedi et al., 2003), verbal short memory (Raz et al., 2007), and auditory memory (Röder et al., 2001). Sensory compensation among the blind has also been observed for some memory-related olfactory tasks. In non-cued odor identification (i.e., free recall of odor names), participants with visual impairments performed better than those without impairments (Murphy and Cain, 1986; Rosenbluth et al., 2000; Wakefield et al., 2004, but see Sorokowska, 2016). In addition, free identification time was shorter among blind compared to sighted individuals (Rosenbluth et al., 2000; Gagnon et al., 2015), highlighting proficiency of visually impaired people in smell-related memory tasks. Further, several studies demonstrated that blind people performed better than sighted individuals in olfactory discrimination, which is often considered to reflect short-term olfactory memory (Cuevas et al., 2009, 2010; Rombaux et al., 2010; Renier et al., 2013; Çomoğlu et al., 2015). However, in the case of this olfactory ability the results were not consistent – other researchers demonstrated that olfactory discrimination skills do not depend on visual status (Schwenn et al., 2002; Beaulieu-Lefebvre et al., 2011; Oniz et al., 2011; Luers et al., 2014; Majchrzak and Eberhard, 2014; Cornell Kärnekull et al., 2016; Guducu et al., 2016; Sorokowska, 2016).

Overall, data suggest that olfactory memory of blind people could be better than that of the sighted individuals. However, a recent study did not confirm this hypothesis (Cornell Kärnekull et al., 2016). Cornell Kärnekull et al. (2016) used a 24-item olfactory episodic recognition test to compare 30 blind individuals aged 26–73 years (*M* = 55.5, *SD* = 12) to a corresponding group of sighted individuals. The participants were further asked to identify a subsample of 12 highly familiar odors. The authors found that episodic odor recognition was similar in blind and sighted subjects. Nevertheless, compensatory effects of visual impairment on olfactory memory might be very complex, as it seems to be the case for olfactory compensation in general (Kupers and Ptito, 2014). Therefore, due to a relatively small sample size, Cornell Kärnekull et al. (2016) could not explore all possibilities in their research. For example, age is an important variable that is related both to olfactory acuity (Sorokowska et al., 2015b) and memory (Choudhury et al., 2003). Choudhury and colleagues measured performance of 231 participants in a 12-item, single-target, four-alternative, forced-choice Odor Memory Test^TM^ (OMT; Sensonics, Haddon Heights, NJ, United States) (Doty, 2003). They observed that age-related decline in performance began around the fifth (i.e., 40–49 years) decade of life. Further, in a study employing 96 subjects tested with 16-item Test for Olfactory Memory (TOM; Croy et al., 2015), olfactory recognition scores of participants older than 60 years were lower than scores obtained by age groups 18–30 and 31–60. However, it is not clear how age affects olfactory memory in blind individuals, as the effect of this variable was not analyzed in the previous study involving this group (Cornell Kärnekull et al., 2016).

Odor identification is a very complex and difficult memory-related task (Chobor, 1992), and the detrimental effects of aging might be due to a decrease in cognitive abilities, which are necessary to correctly identify odor stimuli (Frank et al., 2004, 2011; Hedner et al., 2010). Further, age-related decline in olfactory abilities could be an effect of, among others, diseases (including neurodegenerative problems; Rahayel et al., 2012) or cumulative damage to the olfactory epithelium from repeated infections (Doty, 1989). At the same time, smell training was found to be effective for older adults (Sorokowska et al., 2017). If the superiority of blind people in olfactory abilities indeed results from daily training (Gagnon et al., 2015), their scores should not decrease with age to such an extent as among the sighted people. Here, this hypothesis was tested in a large sample of 94 blind individuals.

## Materials and Methods

### Participants

Hundred and eight sighted individuals (56 women and 52 men) aged 20–64 years (mean age: 38.38 ± 12.12 years) and 94 blind people (48 women and 46 men) aged 16–65 (mean age: 41.70 ± 13.02 years) participated in the study. There were no age statistically significant differences between the groups, although a trend emerged: *F*(1,201) = 3.52, *p* = 0.062.

### Procedure

The study comprised a short interview and assessment of verbal and olfactory memory. During the interview, the participants were questioned about olfactory diseases and overall smell quality. Participants reporting serious olfactory disorders were not included in the subsequent evaluation of olfactory memory. In total, four participants were excluded from further participation in the study (reasons: chronic allergy, facial surgery that involved nose reconstruction, brain surgery, and septum deviation). The participants further completed a verbal retrieval test (one part of DemTect test; Wojtyńska and Szcześniak, 2016).

Olfactory memory was assessed using the TOM (Croy et al., 2015), as it enables testing more than one category of olfactory memory. The TOM comprises episodic odor recognition (discriminating previously presented odors from new odors) and two forms of semantic memory (cued and free identification of odorants), which are tested in three phases. The test is based on 16 smells – anise, pineapple, turpentine, banana, rose, apple, cinnamon, mushrooms, fish, coffee, leather, cloves, peppermint, lemon, garlic, and orange; most items are taken from the basic version of the identification subtest of Sniffin’ Sticks Test, a popular olfactory test based on odor-filled felt-tip pens (Hummel et al., 2007), and mushroom odor is taken from extended version of the Sniffin’ Sticks identification subtest (Haehner et al., 2009; Sorokowska et al., 2015a). In their original study, Croy and colleagues showed that TOM had satisfactory test–retest reliability (*r* = 0.70, *p* < 0.001), and that the time interval between the test and retest sessions did not influence recognition performance in this test. Additionally, odor recognition among subjects with slight cognitive impairment was significantly worse as compared with healthy age-matched controls.

The first phase of the TOM is the Recognition task, in which participants are initially presented with eight target smells and asked to memorize them (the acquisition stage). The odors are presented for approximately 5 s each; presentation interval between the smells is about 15–20 s (Croy et al., 2015). Next, these “old” odors are mixed with eight new smells and presented to the participants again. In this stage of the test (recognition) the participants immediately judge the odors as “old” or “new.” Following signal detection theory, the answers are coded as follows: “old” smells correctly judged as “old” are called “hits,” “old” judged as “new” are called “misses,” “new” judged as “new” are called “correct rejections,” and “new” judged as “old” are called “false alarms.” The second phase of the TOM is the Free Identification task. All 16 odorants are presented to the participants again, and they are asked to identify each smell without any cues. The third phase of the TOM is the Cued Identification task in which the odorants not recognized in free identification task are presented again, but this time the participants are given four response options. For more details of the method and exact instructions see Croy et al. (2015). Additionally, trained research assistants measured response times during the free and cued identification tasks. The measurement was conducted using a stopwatch for each response separately, starting from the moment the participant sniffed an odor (free identification) or heard all response options (cued identification).

The testing was performed in a quiet, well-ventilated room. The whole procedure took about 40 min. This experiment was approved by the Ethical Committee of the Institute of Psychology, University of Wroclaw and it has been carried out in accordance with the guidelines expressed in the Declaration of Helsinki. Written informed consent was obtained from all participants, and they received monetary compensation for their participation. We additionally obtained written parental consent for a few participating teenagers.

### Data Analysis

The following scores were analyzed:

(1) Recognition memory scores in TOM test
  • “Hit,” “false alarm,” “correct rejection,” “miss” rates (scores in each category divided by 8 which were further standardized for analytic purposes)
  • d′ (d prime) scores (calculated as z-score of “False Alarms” subtracted from z-score of “Hits”)
  • response bias (C) (with positive values indicating a conservative response criterion – replying “no” to both old and new stimuli, and negative values indicating a liberal criterion – replying “yes” to both old and new stimuli).
(2) Semantic memory scores in TOM test
  • Free identification score (range: 0–16)
  • Cued identification score (range: 0–16).

In addition to the identification scores, the average free and cued identification response times were calculated for each participant across all correct responses.

(3) Verbal memory – total score in verbal retrieval task (Wojtyńska and Szcześniak, 2016).

We controlled for gender of the participants, as previous studies showed gender differences in olfactory abilities (Doty and Cameron, 2009); importantly, female olfactory superiority was often observed in tasks involving verbal components (Öberg et al., 2002).

**FIGURE 1:**
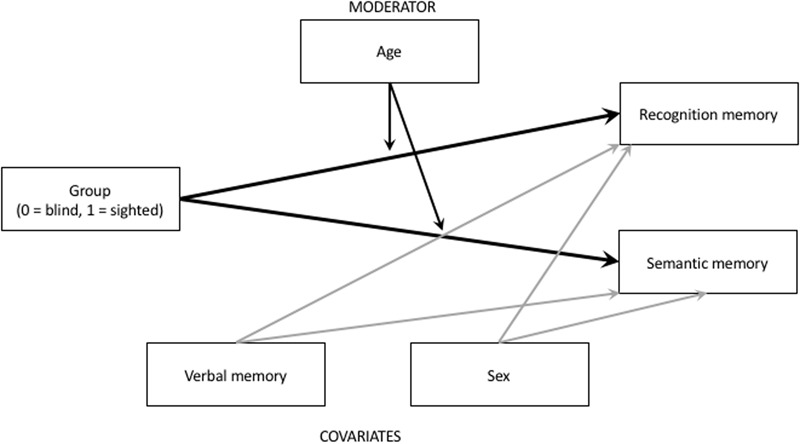
Illustration of the analyzed model.

Data analysis was conducted in a series of multiple regression models, with recognition memory and semantic memory scores being regressed on a group (coded: 0 = blind, 1 = sighted), participants’ age, and theoretically relevant covariates: verbal memory and gender. As there is a possibility that age may modify the difference between sighted and blind individuals, we also included *Group × Age* interaction into our models, to examine possible moderating role played by age. We treated this cross-product effect exploratorily, and we estimated the potential moderating role of age using Johnson-Neyman conditioning technique using Hayes (2013) process macro. Johnson-Neyman formula allows for detection of the curvilinear moderating effects. Conceptually our model is illustrated on **Figure 1**.

## Results

Descriptive statistics and correlations between all variables are presented in **Table 1**. **Table 2** presents the results of the first set of regression models, with recognition memory scores serving as dependent variables.

**Table 1:** Descriptive statistics and intercorrelations between recognition and semantic memory scores.

		Blind													
		*M*	*SD*	1	2	3	4	5	6	7	8	9	10	11	12
1	Hit rate	0.83	0.14	1	0.03	-0.03	–1	0.75	-0.73	0.25	-0.01	0.31	-0.01	0.24	-0.29
2	Correct rejection rate	0.76	0.16	0.25	1	–1	-0.04	0.68	0.66	0.23	-0.14	0.07	-0.22	0.05	0.01
3	False alarm rate	0.24	0.16	-0.26	-0.99	1	0.03	-0.68	-0.66	-0.23	0.14	-0.07	0.22	-0.06	-0.02
4	Miss rate	0.17	0.14	-0.99	-0.25	0.24	1	-0.75	0.73	-0.25	0.02	-0.31	0.01	-0.23	0.30
5	d′	-0.12	1.38	0.77	0.81	-0.82	-0.74	1	-0.10	0.34	-0.10	0.28	-0.15	0.22	-0.20
6	Response bias	0.09	0.67	-0.54	0.67	-0.67	0.55	0.12	1	-0.03	-0.08	-0.18	-0.15	-0.14	0.24
7	Free identification	5.78	2.46	0.32	0.27	-0.28	-0.31	0.38	0.00	1	0.03	0.52	-0.12	0.30	-0.11
8	Free identification time	3.57	2.13	-0.23	-0.17	0.15	0.26	-0.24	0.05	-0.06	1	0.00	0.65	0.09	0.10
9	Cued identification	13.53	2.05	0.48	0.29	-0.29	-0.47	0.48	-0.12	0.46	-0.31	1	-0.06	0.23	-0.01
10	Cued identification time	5.04	2.44	-0.21	-0.17	0.16	0.22	-0.23	0.02	-0.11	0.44	-0.16	1	-0.12	0.27
11	Verbal memory	21.47	4.37	0.16	0.11	-0.13	-0.13	0.18	-0.01	0.32	-0.02	0.32	0.05	1	-0.25
12	Age	41.7	13.02	0.09	-0.23	0.21	-0.07	-0.08	-0.25	-0.08	-0.01	-0.07	0.02	-0.05	1
	Sighted *M*	–	–	0.87	0.75	0.25	0.14	0.13	-0.09	6.13	3.23	14.10	4.24	21.71	38.38
	Sighted *SD*	–	–	0.14	0.19	0.19	0.14	1.64	0.63	2.63	1.89	1.61	2.54	5.47	12.12

*Correlations obtained among blind individuals (*n* = 94) are presented above the diagonal, correlations obtained among sighted individuals (*n* = 108) are given below the diagonal. Correlations equal or higher than *r* = 0.21 above diagonal (and *r* = 0.19 below diagonal) are statistically significant at *p* < 0.05; correlations equal and higher than *r* = 0.27 above diagonal (*r* = 0.25 below diagonal) are significant at *p* < 0.01.*

**Table 2:** A summary of moderated regression models predicting recognition memory scores.

	Dependent variables: recognition memory scores					
Predictors	Hit rate	False alarm rate	Correct rejection rate	Miss rate	d′	Response bias
Group (1 = sighted)	0.27* (0.14)	0.08 (0.15)	-0.11 (0.15)	-0.26ˆ (0.14)	0.14 (0.14)	-0.27* (0.14)
Age	-0.06 (0.07)	0.10 (0.07)	-0.12 (0.07)	0.08 (0.07)	-0.11 (0.07)	0.22* (0.10)
*Group × Age*	0.34* (0.14)	0.27ˆ (0.14)	-0.28ˆ (0.14)	-0.34* (0.14)	0.04 (0.14)	-0.48** (0.14)
Gender	-0.15 (0.13)	0.06 (0.14)	-0.05 (0.14)	0.14 (0.13)	-0.13 (0.14)	0.07 (0.13)
Verbal memory	0.16* (0.07)	-0.10 (0.07)	0.08 (0.07)	-0.13ˆ (0.07)	0.17** (0.07)	-0.05 (0.07)
*R*^2^	0.10	0.04	0.04	0.09	0.06	0.08

*N = 202, Group variable coded: 0 = blind, 1 = sighted, Gender coded: 0 = female, 1 = male. All variables except group and gender were z standardized to facilitate interpretability, so may be read as betas.*

*^∧^*p* < 0.07, ^∗^*p* < 0.05, ^∗∗^*p* < 0.01, ^∗∗∗^*p* < 0.001.*

**FIGURE 2:**
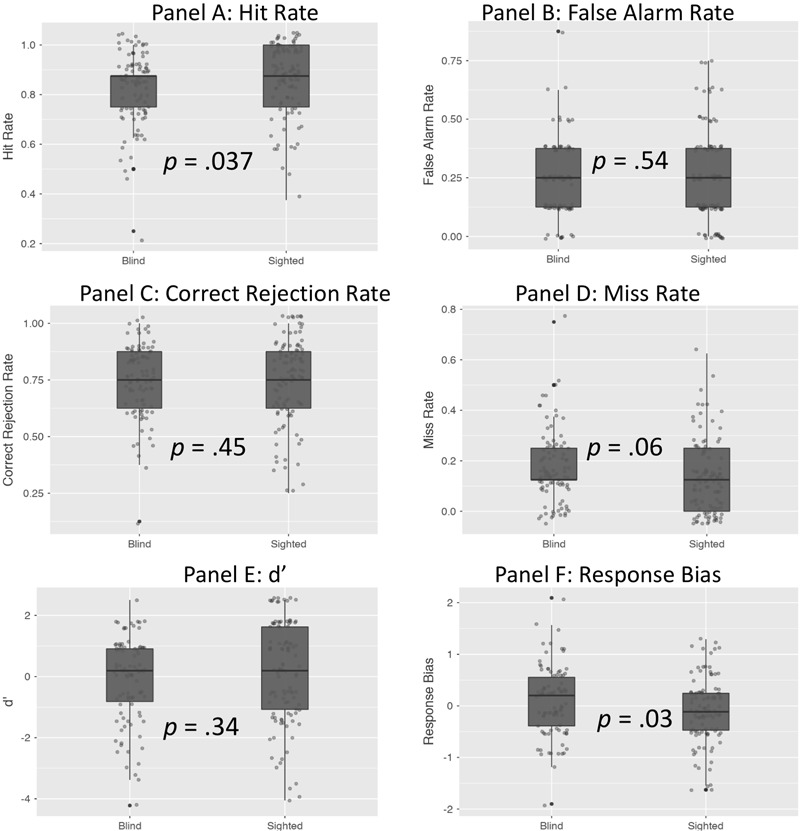
The difference between blind and sighted individuals in recognition memory scores indices.

As illustrated in **Table 2** (see also **Figure 2**), sighted individuals significantly outperformed their blind counterparts in the case of hit rate (*p* = 0.037), and there was a marginal tendency for higher miss rate among blind individuals (*p* = 0.06). Blind individuals’ scores were also characterized by higher response bias (*p* = 0.03). No differences between sighted and blind individuals were observed in the remaining cases – false alarm rate, correct rejection rate and d′ scores were similar in both groups (**Figure 2**).

**FIGURE 3:**
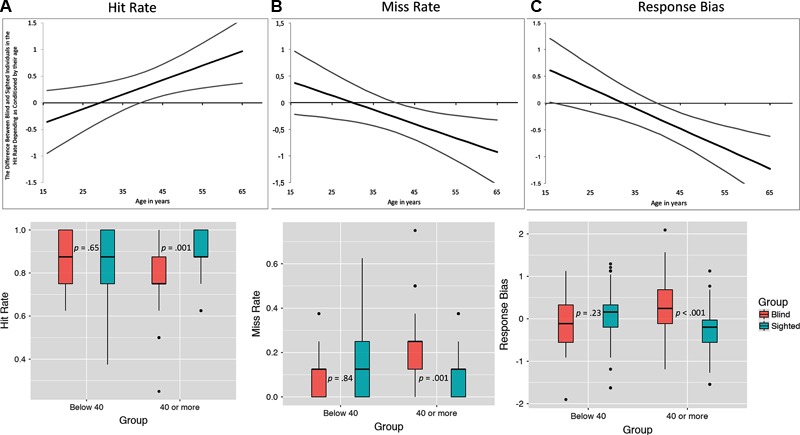
The difference between blind and sighted individuals in hit and miss rates as moderated by their age. Top panels illustrate the zone of significant interaction estimated using the Johnson-Neyman technique. Gray lines are 95% confidence intervals – in the cases if both intervals do not cross X Axis, the moderation effect is statistically significant. Lower panels illustrate the difference between blind and sighted individuals below and above the age levels estimated using Johnson-Neyman method. All values are estimated with the covariates (gender, age, and verbal memory) being partialled-out. Error bars denote standard errors.

It should be noted, however, that age moderated the observed between-group differences in the case of hit and miss rates and response bias. Conditioned moderation using Johnson-Neyman technique (see **Figure 3**), demonstrated that in the case of hit rate, the difference between blind (*n* = 45, *M* = 7.00, *SD* = 0.85) and sighted individuals (*n* = 62, *M* = 6.82, *SD* = 1.22) younger than 40 years old was non-significant, while older sighted individuals (*n* = 46, *M* = 7.09, *SD* = 0.89) outperformed their blind counterparts (*n* = 49, *M* = 6.22, *SD* = 1.25). In the case of miss rate, younger blind and sighted participants did not differ (*M* = 0.98, *SD* = 0.84 and *M* = 1.18, *SD* = 1.22, respectively), yet higher numbers of not recognized odors were observed among blind individuals older than 40 years old (*M* = 1.78, *SD* = 1.25 and *M* = 0.96, *SD* = 0.90, respectively). Similar pattern was observed in the case of response bias – we found no group differences in the case of participants younger than 40 years old (*M*_blind_ = -0.09, *SD*_blind_ = 0.61, *M*_sighted_ = 0.06, *SD*_sighted_ = 0.63, *p* = 0.23), while a significant difference was observed among participants older than 40 years old (*M*_blind_ = 0.25, *SD*_blind_ = 0.68, *M*_sighted_ = -0.28, *SD*_sighted_ = 0.57, *p* < 0.001).

Previous studies exploring the moderating effect of age on olfactory acuity identified the decrease at older age – usually about 55 years (Hummel et al., 2007). For purposes of comparison, we have also examined the potential differences between blind and sighted individuals younger than 55 (*n* = 170) and 55 and older (*n* = 32). In the case of hit rate there were no differences between participants below 55 years of age (*p* = 0.16) and 55 or older (*p* = 0.19). Similarly, miss rates of blind and sighted individuals aged below 55 (*p* = 0.23) and 55 and above (*p* = 0.19) did not differ.

**FIGURE 4:**
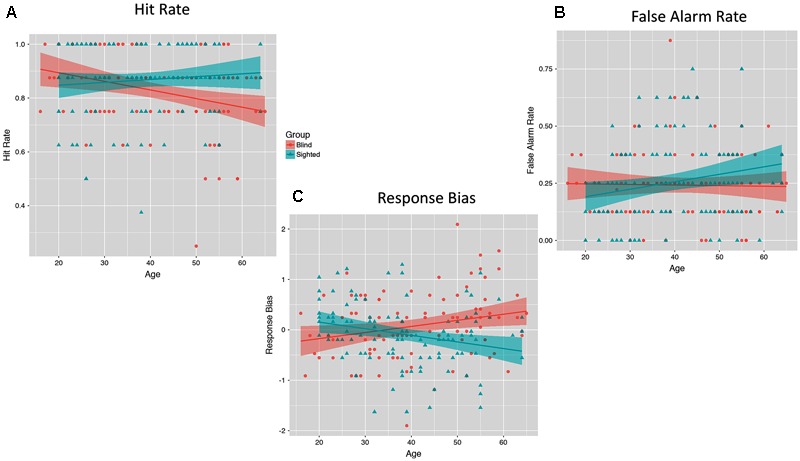
The moderating effect of group (blind versus sighted) on the relationship between participants’ age and hit rate **(A)**, false alarm rate **(B)**, and response bias **(C)**.

Finally, we also plotted links between standardized hit and false alarm rates and age separately for both analyzed groups. As illustrated on **Figure 4**, we observed an age-related decline in hit rate among blind individuals (*B* = -0.25, *SE* = 0.10, *p* = 0.01), while hit scores obtained by sighted individuals were unrelated to their age (*B* = 0.10, *SE* = 0.10, *p* = 0.29). Interestingly, false alarm rate increased with age among sighted individuals (*B* = 0.23, *SE* = 0.10, *p* = 0.023), while it was unrelated to age among blind individuals (*B* = -0.04, *SE* = 0.11, *p* = 0.69). Response bias increased with age among blind individuals (*B* = 0.018, *SE* = 0.01, *p* = 0.025), while it decreased with age among sighted individuals (*B* = -0.02, *SE* = 0.01, *p* = 0.01).

The model illustrated on **Figure 1** was also applied to semantic memory scores (**Table 3**). Blind and sighted individuals did not differ in free identification score and its response time, but sighted individuals outperformed blind individuals in cued identification score (*p* = 0.03). The (marginal) difference (*p* = 0.056), was also observed in the case of cued identification response time: blind individuals responded in a slower manner than sighted individuals. No age-moderated effects were observed.

**Table 3:** A summary of moderated regression models predicting semantic memory scores.

	Dependent variables: semantic memory scores			
Predictors	Free identification	Free identification response time	Cued identification	Cued identification response time
Group (1 = sighted)	0.12 (0.14)	-0.15 (0.14)	0.30* (0.14)	-0.27ˆ (0.14)
Age	-0.04 (0.10)	0.11 (0.10)	0.05 (0.10)	0.25* (0.10)
*Group × Age*	-0.03 (0.14)	-0.10 (0.10)	-0.09 (0.14)	-0.22 (0.14)
Gender	-0.01 (0.14)	0.36* (0.14)	0.10 (0.14)	0.19 (0.14)
Verbal memory	0.30*** (0.07)	0.05 (0.07)	0.27*** (0.07)	0.01 (0.07)
*R*^2^	0.10	0.05	0.10	0.06

*N = 202, Group variable coded: 0 = blind, 1 = sighted, Gender coded: 0 = female, 1 = male. All variables except group and gender were z standardized to facilitate interpretability, so may be read as betas.*

*^∧^*p* < 0.07, ^∗^*p* < 0.05, ^∗∗^*p* < 0.01, ^∗∗∗^*p* < 0.001.*

## Discussion

In the current study, we tested the hypothesis regarding assumed superiority of blind people over sighted individuals in the area of olfactory memory. We also analyzed the potential age-related effects with this regard. In terms of episodic olfactory memory, we observed an age-related decline in correct hits in blind participants, but an age-related increase in false alarms in sighted participants. Interestingly, in sighted individuals, the increase in false alarms reflected a change in response bias – these participants adopted a more liberal response criterion as they grew older. Semantic memory scores in both blind and sighted individuals were not affected by their age. Further, we found that sighted individuals outperformed their blind counterparts in the case of some TOM test subscores (hit rate, miss rate, and cued identification score). Age moderated the between-group differences for hit and miss rates, but the direction of the observed effect was contrary to our expectations. The difference between blind and sighted individuals younger than 40 years old was non-significant, while older sighted individuals outperformed their blind counterparts. Nevertheless, in most TOM test subscales we found no differences between sighted and blind individuals.

Numerous studies showed deterioration of smell in elderly people (Doty, 1989; Hummel et al., 1998; Murphy et al., 2002; Sorokowska et al., 2015b), and as expected (Choudhury et al., 2003), age proved to be a significant factor also in our analyses of olfactory episodic memory. However, contrary to our assumptions, olfactory memory scores of older blind people were not higher than these of older sighted people. We even noted superiority of sighted participants in two TOM subtests among individuals older than 40 years (although this effect disappeared when we compared people 55 and older – this, however, may be caused by a limited statistical power of this specific comparison). In the context of previously discussed studies on olfactory abilities of blind people, the hypothesis regarding an automatic increase of olfactory abilities among blind people due to daily smell training (Gagnon et al., 2015) finds no support in our findings.

However, it needs to be noted that we did not observe an age-related decline in semantic memory scores in our sample. Perhaps, consistent with previous studies on smell identification performance, we would need to include a larger sample of individuals older than 50 years to conduct more detailed analyses (Zhang and Wang, 2017), especially that healthy, normosmic subjects normally perform very well in olfactory identification tests (Hummel et al., 1997, 2007). The ceiling effect in the case of cued identification in the case of cued identification could have obscured any existing inter-group differences and it might have reduced the likelihood of observing a significant sensory compensation effect for semantic olfactory memory.

Overall, our results are in agreement with the findings of Cornell Kärnekull et al. (2016) on olfactory memory. Nevertheless, the discrepancy between our results and the previous literature on olfactory abilities of blind people is very interesting. There are several factors that could have contributed to this difference. First, researchers studying olfactory performance of blind people use various methods, and – independent of any possible sensory compensation – it is possible that some tests (like the TOM test; Croy et al., 2015) are not equally easy for sighed and blind individuals. For example, olfactory memory depends to a great extent on previous knowledge of applied odorants (Cornell Kärnekull et al., 2015), and familiarity is crucial for identification test performance (Hummel et al., 1997). Relatedly, smell identification tests need to be standardized and adapted to be used in new cultural settings (Oleszkiewicz et al., 2016). As Sniffin’ Sticks identification test (Hummel et al., 2007) was standardized in a sighted population, it is possible that blind people are less familiar than sighted individuals with odors comprising this test and – consequently – the TOM test (Croy et al., 2015). Further, there could be certain sample differences between the present and previous studies that found enhanced olfactory performance in the blind. Some authors explain the olfactory superiority of the blind people in terms of higher attention to olfactory stimuli (Ferdenzi et al., 2010). For example, blind people could use olfactory cues to develop spatial representations (Espinosa et al., 1998). This increased perceptual attention might result in deeper knowledge of odors and a better ability to reactivate associated information, although not all blind people need to be equally smell-oriented. Future studies should not only analyze the effect of blindness on various aspects of olfactory acuity, but also take into account the self-assessed attention people pay to olfactory cues.

Olfactory identification (a domain of semantic odor memory analyzed in the current study) was speculated to be a result of, among others, general semantic knowledge and verbal skills (Larsson et al., 2000, 2004; Frank et al., 2004; Hedner et al., 2010). Original TOM test paper demonstrated decreased scores among demented individuals, which suggests a high impact of cognitive factors on performance in this test (Croy et al., 2015). Our study concurs with these findings – we found that verbal memory test scores were positively related to hit rate, d prime score and free identification abilities among all participants. This is probably due to similar structure (White, 1998) and dynamics (Cornell Kärnekull et al., 2015) of olfactory memory and other memory systems. As both in our and the previous study (Cornell Kärnekull et al., 2016) these results were very similar for blind and sighted people, it seems that performance in odor memory test depends on variables other than visual impairment and sensory compensation.

Previous studies suggest that women tend to have better episodic olfactory memory than men, and their better performance is likely mediated by their higher proficiency in odor identification (Öberg et al., 2002). In addition, olfactory superiority of female participants was found to depend on verbal abilities (Larsson et al., 2003), as activating verbal information is crucial for recognition of odors. However, in the current study we found no effects of participants’ gender. This might have resulted from low familiarity of odorants applied in our research, as indicated by relatively low free identification scores in our samples. Performance in memory-related olfactory tasks can rely on prior exposure to and familiarity with the target odors (Richardson and Zucco, 1989; Öberg et al., 2002; Cornell Kärnekull et al., 2015). Future studies could additionally involve individual assessments of familiarity of each odor, as low familiarity of applied odorants can significantly decrease olfactory memory (Cornell Kärnekull et al., 2015).

## Conclusion

We found no effect of visual impairment on olfactory memory. Although blindness can be a driving force behind a variety of changes in sensory systems, we did not observe sensory compensation among the blind in several olfactory-related memory tasks. In addition, we found that olfactory memory of older sighted participants was higher compared to blind participants, which suggests that daily perceptual training is not enough to enhance olfactory function.

## Author Contributions

Contribution of AS and MK: substantial contributions to the conception or design of the work; or the acquisition, analysis, and interpretation of data for the work; drafting the work or revising it critically for important intellectual content; final approval of the version to be published; agreement to be accountable for all aspects of the work in ensuring that questions related to the accuracy or integrity of any part of the work are appropriately investigated and resolved.

## Conflict of Interest Statement

The authors declare that the research was conducted in the absence of any commercial or financial relationships that could be construed as a potential conflict of interest.
